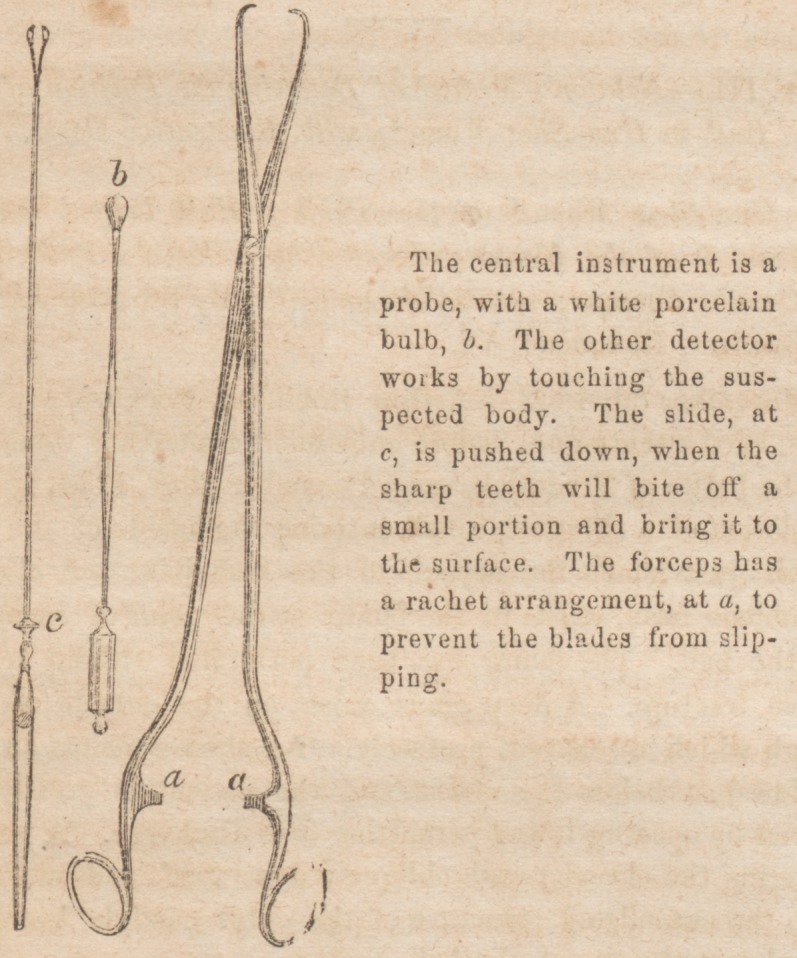# Nelaton's Method of Detecting the Position of the Ball in Gun-Shot Wounds, with Illustrative Cases—i. Gun-Shot Wound of the Thigh; Ball Lodged in the Condyle of the Femur; Detected by Nelaton's Probe and Canula Forceps; Successful Removal;—ii. Letter to the Editor from Surgeon A. Y. P. Garnett, on the Use of Nelaton's Probe

**Published:** 1864-02

**Authors:** James Bolton

**Affiliations:** Richmond, Va.


ART. III.—*Nelaton's Method of Detecting the Position of the Ball in Gun-Shot Wounds, with Illustrative Cases.*

1.—*Gun-Shot Wound of the Thigh; Ball lodged in the Condyle of the Femur; detected by Nelaton's Probe and Canula Forceps; successful removal.* By Surgeon JAMES BOLTON, Richmond, Va.

November 3d, 1863.—Arthur Robinson, Rockbridge artillery, aged twenty-one; before enlistment a student; wounded at the battle of Fredericksburg, December 20th, 1862, by the explosion of a shrapnel, a ball entering the inside of the left thigh, just above the capsule of the knee-joint. A cloaca, of oblong form, exists in the femur at the point of entrance of the ball. The femur has been perforated without transverse fracture. A deep sinus exists on the outside of the thigh which discharges profusely. An abscess points on the inside just below the cloaca, and the patient is greatly relieved by opening it and permitting it to discharge. A probe, entering the cloaca, passes obliquely downwards and outwards into the cancellated structure of the outer condyle, but does not detect the site of the ball.

The patient suffers considerably from constitutional irritation, producing loss of flesh, irritable pulse and fever. The knee-joint has not been much affected and its function is almost unimpaired.

Nov. 5th—Operation.—Passed Nelaton's probe through the cloaca, and, after searching the interior of the cavity, at length discovered distinct marks of lead upon the porcelain. Then explored with the canula forceps and nipped off a small piece of lead, having bright surfaces as if freshly cut with a knife. The evidences furnished by these explorations were beautifully distinct and satisfactory. They reminded me of the results of deep-sea soundings, when the lead brings up samples of the debris composing the bed of the sea. The position of the ball having thus been detected with precision, it was seized with the forceps, and, after repeated and powerful efforts, it was dislodged from its bed, where it lay firmly impacted in the cancellated structure of the outer condyle of the femur. On attempting to extract the ball, the cloaca was found to have contracted to such a degree as to bar its exit completely. The aperture was then enlarged by means of a carpenter's brace and counter-sink bit, and was still further enlarged by a chisel and hammer. The ball was then extracted without difficulty.

The operation, including the explorations, occupied an hour and a half, during which time the patient was kept steadily under the influence of chloroform.

This case is one of unusual interest. During the eleven months which intervened between the receipt of the wound and the operation, the ball had frequently been searched for, but no positive opinion had been arrived at in regard to its exact site; nor do I know any means by which the doubt and obscurity could have been removed, except those used. It was, therefore, a perfect and triumphant success.

The central instrument is a probe, with a white porcelain bulb, *b*. The other detector works by touching the suspected body. The slide, at *c*, is pushed down, when the sharp teeth will bite off a small portion and bring it to the surface. The forceps has a rachet arrangement, at *a*, to prevent the blades from slipping.

2.—*Letter to the Editor from Surgeon* A. Y. P. Garnett, *on the Use of Nelaton's Probe.*

Dear Sir,—In compliance with your request, I herewith furnish you with a brief statement embracing the results of my experience with the use of Nelaton's probes. These highly useful, but simple and ingenious little instruments, I am informed, were invented by this eminent French surgeon for the purpose of exploring a gun-shot wound received by the notorious Garibaldi at the time of his capture. The ball having buried itself in some portion of the foot, it was found impossible, by any of the ordinary methods of exploration, to distinguish it from the bony structure into which it had penetrated.

In this condition the patient was conveyed to Paris and placed under the professional care of Nelaton, whose operative skill and brilliant genius has achieved for him so widespread and distinguished a reputation.

Through the instrumentality of these probes, which he devised for the occasion, he was enabled to discover the exact locality of the ball and succeeded in removing it from the foot. My attention was first called to these instruments by Surgeon James Bolton, who had procured them from the office of the Surgeon-General, with the view of exploring a gun-shot wound of the thigh of long standing and involving the femur. All preceding investigations having failed to discover the exact position of the ball and the extent of injury to the bony structure implicated, I assisted in the examination of the case, and was surprised, as well as gratified, at the accuracy with which we detected the ball deeply imbedded in the cancellated structure of the external condyle. The reliability of the instruments was put to a rigid test in this case by the mutually corroborative evidence of each, the porcelain bulb exhibiting clearly the metallic impression, and the canulated nippers unmistakably confirming the diagnosis by bringing away small portions of the metal.

The details of this case, I believe, have been already presented to you by Surgeon Bolton, who had immediate charge of the patient.

Case No. 2 was that of Lieutenant R., who, whilst practising at a pistol gallery in firing with a duelling pistol, accidentally shot himself through the metatarsal bones of the right foot. I was called to this case a short time subsequent to the receipt of the wound, but was unable to discover the position of the ball by the use of the ordinary silver probe or my finger. There was very slight hemorrhage and the patient was put to bed; a full anodyne exhibited and cold cloths applied to the wound. He was soon after conveyed to the residence of Professor Gibson, who was at the time absent from the city. Some three weeks after, at the request of Dr. Gibson, I was present and assisted in a thorough examination of the wound, with the determination, if practicable, of finding and extracting the ball. In passing the probe down the track of the ball, it soon came in contact with a hard substance, which the doctor seemed inclined to believe might be the ball, but which we had no way of distinguishing from the bony structure, unless by cutting down through the soft parts to the point in doubt. My own impression was that the ball had passed through the bony arch of the foot, but, as neither of us had found it practicable to pass the probe through to the soft parts beneath, I was forced to acknowledge the probable fallibility of such a conclusion. It was finally determined to submit the case to the diagnostic elucidation of Nelaton's probes. These having been procured, we first passed down upon the solid body, previously alluded to, the porcelain bulb probe, and making pressure, whilst giving it a rotary motion for several seconds, we removed it, and found that no metallic impression had been left upon it. This operation was repeated with this probe several times and with similar results in each instance. We next tried the nippers, and discovered that they contained, when removed, a small piece of bone. Taking the results of these two examinations in connexion, the one substantiating or verifying the negative evidence of the other, we were forced to adopt the opinion, that there was no ball at that point, but that it had passed through the bony arch of the foot and lodged somewhere in the subjacent tissues. Acting upon this conclusion, although the most careful manipulation had failed to indicate the existence of a foreign body at all, an incision was made through the soft parts beneath and immediately opposite the wound upon the dorsum of the foot, sufficiently large to admit the introduction of the index-finger, with which the ball was readily felt lying beneath and in contact with the metatarsal bones. It is scarcely necessary to add that the ball was removed and that the patient recovered the entire use of the foot.

Case No. 3 exhibits in perhaps a still more gratifying manner the important and useful agency of these instruments. On the 18th of December, 1863, Mr. C. was brought to the Robertson Hospital of this city suffering with a gun-shot wound of the right thigh, received some six or more months prior to his admission; the ball having entered the external part of the thigh, about the junction of the upper with the middle third, and ranged obliquely inwards and upwards. He stated that he had suffered with severe constitutional symptoms, his general health having undergone a gradual and almost uninterrupted decadence since the receipt of the wound; that the wound had been repeatedly examined by different medical officers, but that on no occasion had any one of them been able to ascertain the existence or position of the ball, which still remained somewhere concealed in the limb At the time of his entrance into the hospital, his general health had somewhat improved, although he presented a pallid and feeble appearance; he was unable to use the wounded extremity at all, and was scarcely able to get along with the aid of crutches. The wound was still discharging slightly, but presented a small red spot not much larger than a garden pea. There was also an indurated condition of the tissues along the anterior aspect of the limb and somewhat circumscribed, which, though not painful to the touch, had given rise to the suspicion that a large abscess was about to develope itself at that point.

Having procured a long gun-shot probe, I passed it along the track of the ball until it impinged against the upper surface or periphery of the thigh bone. With some difficulty and perseverance I finally succeeded in passing the probe beyond this obstruction, obliquely inwards and upwards, towards the adductor muscles, until it came in contact with a hard, rough body, which seemed to be lying immediately contiguous to the femoral artery. Taking into consideration the history of this case, I was somewhat puzzled to determine whether this body was the ball or a fragment of bone which had been chipped off at the time the wound was inflicted, when it occurred to me that this important problem might at once be solved by the unerring test of Nelaton's probes. I was not disappointed in my anticipated triumph; for, notwithstanding some difficulty experienced in consequence of the insufficient length of the porcelain bulb, I succeeded in obtaining the metallic mark at several different exploratory operations with it. A few days after, assisted by Surgeon Bolton, the patient, having previously been placed under the influence of chloroform, an incision was made, about three inches long, through the external wound, down to the bone, and the track of the ball beyond enlarged with a blunt-pointed bistoury, to avoid the possibility of wounding the femoral artery. Through this, an ordinary pair of bullet forceps was introduced and the ball extracted. The patient had no bad symptoms whatever; the wound healed almost entirely at the expiration of three weeks, when he was permitted to return to his home on furlough.

The above embraces the only cases in which I have had an opportunity of employing these probes; but, limited as my experience has been, it has demonstrated conclusively to my mind the important advantages secured to the military surgeon by this useful invention, and I take pleasure, as an humble member of our profession, in expressing my high appreciation of its merits, and, at the same time, in acknowledging the obligations under which the profession has been placed to the distinguished inventor.

It is, perhaps, as well that I should state in this connexion, that I have experienced great difficulty in removing from the porcelain bulb the particles of metal, in order that it may be fit for future use—having failed with warm water, soap and brush, acids and other agents. It seems that it will be necessary to subject these metallic particles to some chemical process, by which the metal may be oxidized and formed by the agency of an acid into some of the soluble salts of lead. Of this, however, you are far more competent to judge than I am.

[Nitric acid in excess, or acetic acid will cause oxydation of the metal and the formation of a soluble salt.—Ed.]

## Figures and Tables

**Figure f1:**